# Tick-borne *Rickettsia, Anaplasma, Theileria*, and enzootic nasal tumor virus in ruminant, PET, and poultry animals in Pakistan

**DOI:** 10.3389/fmicb.2024.1359492

**Published:** 2024-03-26

**Authors:** Anjum Jamil, Ze Yu, Yuxin Wang, Qing Xin, Shan Gao, Muhammad Abdul Wahab, Xiaohu Han, Zeliang Chen

**Affiliations:** ^1^Key Laboratory of Livestock Infectious Diseases, Ministry of Education, Shenyang Agriculture University, Shenyang, China; ^2^Department of Epidemiology, School of Public Health, Sun Yat-sun University, Guangzhou, China; ^3^Department of Veterinary Parasitology, University of Agriculture Faisalabad, Faisalabad, Pakistan

**Keywords:** enzootic nasal tumor virus (ENTVs), *Theileria*, *Anaplasma*, *Rickettsia*, TBPs

## Abstract

**Introduction:**

Pakistan is an agricultural country; most of its income is based on livestock rearing. The increasing prevalence of tick-borne pathogens among animals may affect the animal production and livelihood of owners, which eventually derange the economy of a country.

**Methodology:**

To further comprehend TBPs, 213 ticks were collected from different animals, including ruminants, pets, and poultry. After molecular and phylogenetic analysis identification, ticks were managed into different pools based on their species level (*Hyalomma anatolicum* = 80, *Rhipicephalus microplus* = 35, *Hyalomma scupense* = 23, *Rhipicephalus turanicus* = 70, and *Rhipicephalus sanguineus* = 5).

**Results and discussion:**

After tick species identification, further molecular PCR amplification was carried out to screen out the pathogens for the presence of *Theileria, Rickettsia, Anaplasma*, and enzootic nasal tumor virus (ENTV). The following pathogens were detected: 11 (5.16%) for *Anaplasma*, 1 (0.47%) for *Rickettsia*, and 9 (4.23%) for *Theileria*. Nevertheless, other TBPs that had not been reported so far in Pakistan 3 (1.41%), were positive for enzootic nasal tumor virus (ENTV). Besides, phylogenetic analysis of the enzootic nasal tumor virus (ENTV) strain confirmed its resemblance to the Chinese strain, while Anaplasma has comparability with Pakistan and China, *Rickettsia* with Pakistan, China, and Iran, and *Theileria* with India, South Africa, United States, Japan, and Spain.

**Conclusion:**

This study reveals that there is a considerably wider range of TBPs held in Pakistan that take in various contagious zoonotic pathogens than was previously thought. This information advances TBP epidemiology and will contribute to upgrade future control measure.

## 1 Introduction

Pakistan is an agricultural country with a profitable environment for the production of livestock. Various livestock species are sheep, goats, cattle, and buffalo, with populations of 28.8, 64.9, 38.3, and 33.7 million, respectively. Sheep play a pivotal role in the dairy sector in Pakistan, following China and India, which have consequential goat and sheep-raising sectors. The financial results of bringing up livestock in Pakistan fail to achieve their maximum potential (Jabbar et al., 2015). Among the vector-borne diseases, the more bonafide complications emanate due to tick-borne disease, which is prevalent in the world and may lead to severe complications. It has been reported that about 1 million deaths are observed annually due to tick-borne pathogens (WHO, 2014). Ticks are an immense source of spreading zoonotic diseases such as tularemia, tick-borne relapsing fever, Rocky Mountain spotted fever, anaplasmosis, and ehrlichiosis (Bratton and Corey, 2005; Hussain N. et al., 2023). Developing countries like tropical and subtropical countries are in consensus due to TBDs exerting economic challenges on the worldwide livestock sector. Piroplasmosis is the protozoal disease caused by *Babesia* and *Theileria*, although anaplasmosis and rickettsiosis are caused by *Anaplasma* and *Rickettsia*, respectively, and have consequential prestige in the dairy sector. Ruminants are highly vulnerable to tick-borne pathogens. The most commonly reported diseases are anaplasmosis, theileriosis, and piroplasmosis, which affect the reproductive status of the livestock population (Saeed et al., 2015; Sajid et al., 2017; Khan et al., 2022).

ENA is a tumor of sheep and goats, and it is chronic and contagious in nature. It is summarized as the proliferation and thickening of the lining of secretory epithelial cells in the nasal cavity (De las Heras et al., 2003; Svara et al., 2006; Özmen et al., 2010). This tumor is caused by ENTV, a retrovirus, an enzootic nasal tumor virus in small ruminants (Leroux and Mornex, 2008). The retroviruses are highly complicated in structure and are mostly RNA viruses, which are distributed in various vertebrate and non-vertebrate species. Some instances include the small ruminant jaagsiekte sheep retrovirus (JSRV), lentiviruses, and the most contagious ENTV, which cause infections in sheep and goats. ENA was first reported in Germany in 1939 and, in due course, proclaimed in all major livestock farming sectors, especially sheep and goat rearing sites (De las Heras et al., 1995). The tumor is widespread globally and has been identified across many countries, particularly in Turkey, in recent years (Özmen and Serpin, 2016). Pathogenesis of ENTV is similar to another tumor-causing beta retrovirus, JSRV, which causes respiratory issues such as pulmonary adenocarcinoma in sheep and goats (Monot et al., 2015). Both ENTVs have two types in nature: ENTV 1 affects goats and ENTV 2 affects sheep. Both have drastic effects and may cause severe complications (Cousens et al., 1999; Ortín et al., 2003; Bratton and Corey, 2005; WHO, 2014), such as depression, anorexia, exudative nasal discharge with bleeding, and difficulty breathing may lead to stretching the neck, eventually facial complications, and even death due to chronic respiratory blockage (De las Heras et al., 1995; Walsh et al., 2013).

Pakistan's subtropical geography provides a perfect environment for the growth and proliferation of tick populations, which are significant testimony to hemoparasitic diseases. Various tick species, particularly *Boophilus, Ixodidae, Hyalomma*, and *Rhipicephalus*, have a consequence role in imparting hemoparasitic diseases (Eshetu, 2015), with *Hyalomma* species being the most abundant in Pakistan (Atif et al., 2012).

Most etiological surveys diligently use conventional standard methods, such as reviewing stained blood smears under a microscope. Regardless of its affordability, this approach requires technical proficiency and may lack specificity when addressing morphologically similar TBPs that coexist. In addition, its sensitivity is limited in cases of low parasitemia. To date, there has been no confirmation of tick-borne ENTV in Pakistan. Prior research was defined to certain geographic areas and overlooked to contemplate agroecological areas, methods of production, and sample strategies. These factors can greatly impact the solidity of estimates of the prevalence of diseases transmitted via ticks. The lack of accurate data on the epidemiology of tick-borne diseases (TBDs) makes it difficult to map current affluence as well as the distribution of tick-borne pathogens (TBPs) in Pakistan.

This study made an effort to locate the tick-borne pathogens circulating in Pakistan due to the limited molecular diagnostic-based information available on their epidemiology. Our results suggest the prevalence of *Anaplasma, Theileria*, and *Rickettsia* among livestock populations in Pakistan. Furthermore, we reported ENTV for the first time from ticks of sheep and goats, where small ruminant farming is highly pronounced. These findings may expedite the development of various productive strategies for controlling TBDs in Pakistan.

## 2 Materials and methods

### 2.1 Study area

**Figure 1:**
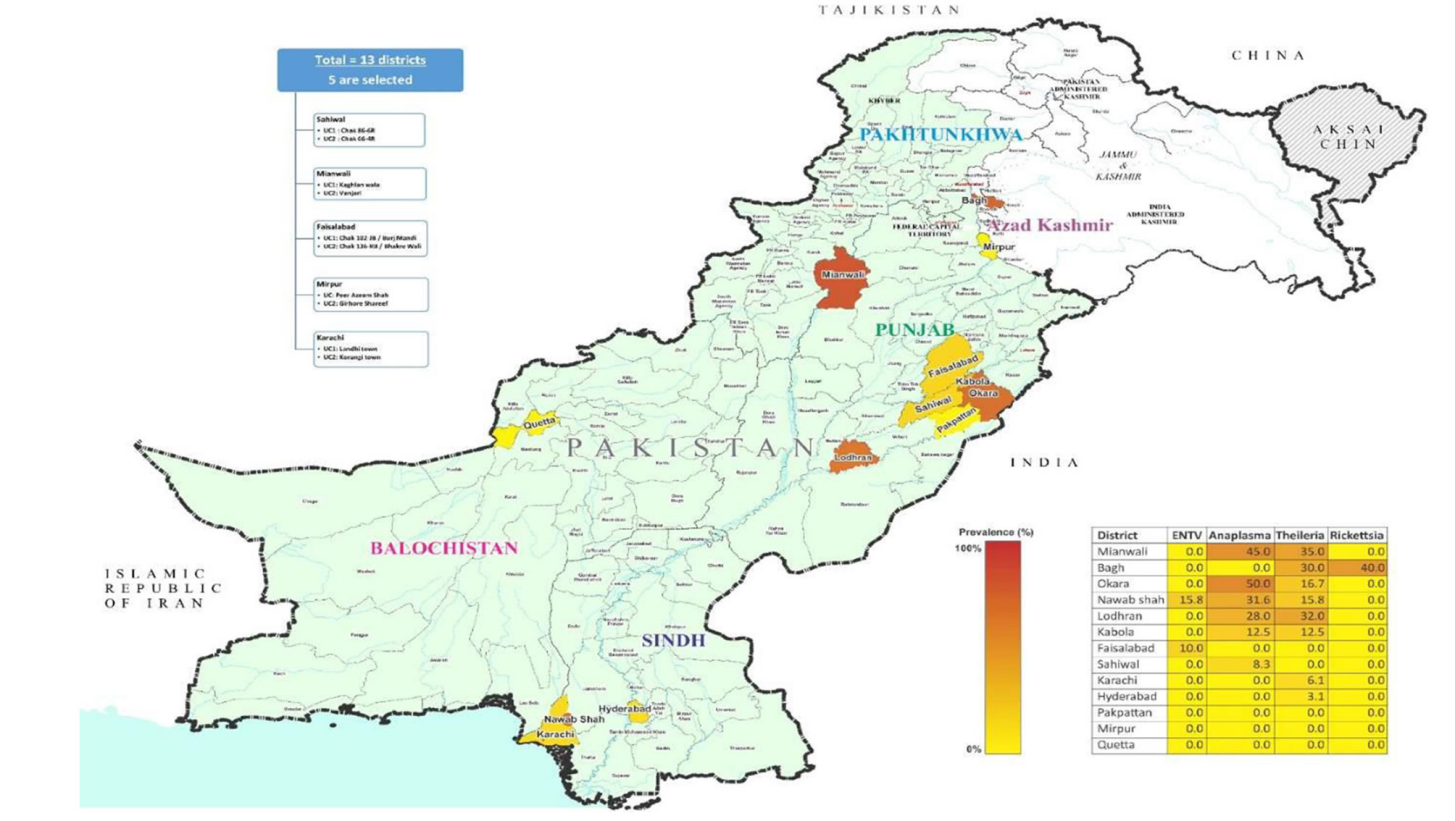
Location and distribution of the study area.

To expedite the ticks collection, 13 districts were selected based on livestock population, including Sahiwal (30.677717° N, 73.6245232'°E), Okara (30.8138°N, 73.4534°E), Faisalabad (31.4504° N, 73.1350° E), Pakpattan (30.34314° N, 73.38944° E), Kabola (30.1763° N, 73.0700° E), Lodhran (29.5339° N, 71.63244° E), Mianwali (32.5839° N, 71.5370° E) of Punjab, Karachi (24.8607° N, 67.0011° E), Hyderabad (17.4065° N, 78.4772° E), Nawabshah (36.2447° N, 68.3935° E) of Sindh, Bagh (33.9794° N, 73.7772° E), and Mirpur (33.1480° N, 73.7537° E) of Kashmir and Quetta (30.1798° N, 66.9750° E) of Balochistan from Pakistan. The average temperature of Punjab districts is 5–15°C and 40–50°C, while the Sindh districts are 10–20°C and 30–50°C. In the case of Balochistan districts, 25–35°C and 5–18°C, and in Kashmir districts, 10–20°C and 2–15°C in winter and summer seasons, respectively. Tick specimens were collected from a variety of hosts that were present in easily accessible areas of the research location. Using a Global Positioning System (GPS), Figure 1 illustrates the location and distribution of the study area.

### 2.2 Study design

**Figure 2:**
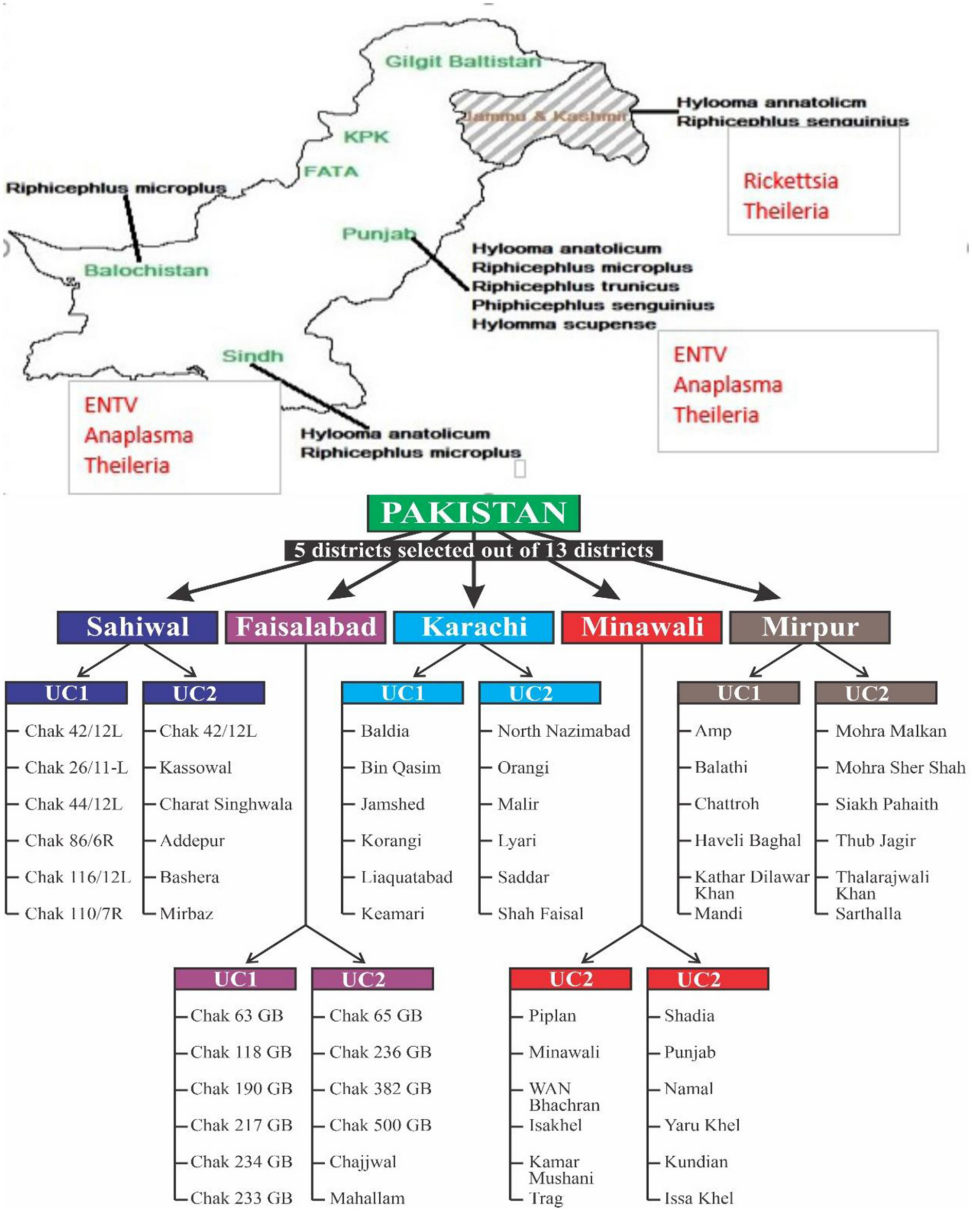
Study design.

A cross-sectional research investigation was conducted to explore the prevalence and geographic distribution of ticks infesting ruminant species on livestock farms. The sample size was calculated based on substantial populations, considering a prevalence of 50% with a 95% confidence level and a desired precision of 10%. At least 70 dairy farms need to be sampled according to this data processing (Jabbar et al., 2015). The number of animal farms was raised to 105, based on the administrative units. In the initial phase, five districts, which accounted for 25% of the total 32 districts, were chosen. Figure 2 shows the district and its relevant villages for tick sampling and pathogen burden.

### 2.3 Tick collection

Tick surveys were conducted on domestic animals in 2021–2022. Tick samples were collected during the tick active period of March–May and August–October. In a simple cross-sectional survey, we studied goats (*n* = 51), sheep (*n* = 44), cattle (*n* = 43), buffalo (*n* = 46), poultry (*n* = 5), dogs (*n* = 21), and cats (*n* = 3) among domestic animals. The assessed animals were traditional livestock from nomadic and local small farms with varied housing systems and had never been vaccinated or parasitized. The animals' ears, udder, scrotum, tail base, neck, and chest were checked for ticks. Ticks on small and large ruminants took 3–5 and 7–15 min to be found, respectively. All ticks were placed in 2 ml vials. For further laboratory work, ticks were preserved at −80°C. Ticks specimens were collected in glass vials with 70% ethyl alcohol and taken to the parasitology lab for identification. Morphological keys identify tick species under a stereomicroscope (Jamil et al., 2023).

### 2.4 Tick genomic DNA extraction

After the preservation of ticks, further tick DNA was extracted from morphologically recognized ticks (20 per species). The ticks were taken and dissected horizontally into segments for sample analysis. The tick sample was cut into six different sections and placed in a Falcon tube filled with RNA-free water for extraction. The tick fragments were washed three times with RNA-free water. Tick specimens of each species were cut into pieces using a new scalpel blade and then placed in a 1.5-ml microcentrifuge tube for DNA extraction using the manufacturer's kit instructions.

### 2.5 Polymerase chain reaction for 16s rRNA amplification

After DNA extraction, further PCR amplification was carried out by amplifying the ~460-bp fragment of the 16S rRNA gene by managing with a set of primers (forward) 5′-CTGCTCAATGATTTTTTAAATTGCTGTGG-3′ and (reverse) 5′-CCGGTCTGAACTCAGATCAAGT-3′. PCR reactions were performed in a 30-μl reaction mixture, including 2.5 μl of genomic DNA, 1 μl of each primer (10 pmol), 10.5 μl of PCR grade water, and 15 μl of master mix. PCR reactions were conducted with the following parameters: initial denaturation at 95°C for 5 min, followed by 40 cycles of denaturation at 95°C for 45 s, annealing at 50°C for 45 s, extension at 72°C for 1 min, and final extension at 72°C for 5 min.

### 2.6 Gene amplification for detection of *Theileria, Rickettsia, Anaplasma*, and ENTV

#### 2.6.1 PCR amplification of *Theileria*

PCR amplification was implemented to ascertain the *Theileria* in tick DNA, following base pair 426–430 of the 18S rRNA gene of *Theileria*, which was targeted through the use of previously reported primers (Mwamuye et al., 2017): (forward) 5′-CACAGGGAGGTAGTGACAAG-3′ and (reverse) 5′-AAGAATTTCACCTCTGACAG-3′. The master mixture for the PCR volume mixture is composed of 25 μl (Taq polymerase enzyme, 0.5 μl; PCR buffer, 2.5 μl; extracted DNA, about 3 μl; primers, 1 μl; double distilled H_2_O, 15 μl; dNTPs, 0.5 μl; and MgCl_2_, 1 μl). After preparation of the volume, PCR amplifications were performed with the following conditions: the first step of initial denaturation lasted for 5 min at 95°C, followed by 30 cycles of denaturation lasting for 15 s at 95°C, followed by primer annealing lasting for around 15 s at 54°C, and two extensions lasted from 30 s at 72°C to 5 min at 72°C.

#### 2.6.2 PCR amplification of *Anaplasma*

A 309-base pair fragment from the 16S rRNA gene of *Anaplasma* was targeted using previously described primer sequences (Xu et al., 2011): (forward) 5′-GGTACCYACAGAAGAAGTCC-3′ and (reverse) 5′-TAGCACTCATCGTTTACAGC-3′. After having a set of primers, the volume of the master mixture and chemicals used are kept in the same proportion as discussed in the *Theileria* section. After making the volume, the PCR protocol was set up as follows: initial denaturation was performed for 5 min at 95°C, followed by denaturation for about 15 s at 95°C, subsequently annealing for primer lasted for 15 s at 54°C, and finally, two extensions were performed for 30 s at 72°C and 5 min at 72°C.

#### 2.6.3 PCR amplification of *Rickettsia*

To detect *Rickettsia*, conventional PCR primers targeting the citrate synthase gene (*gltA*) of 1,178 bp were used (Xue et al., 2023). The reaction mixture is composed of 25 μl (almost 1 μl of MgCl_2_, 0.5 μl of enzyme, 3 μl of extracted DNA, moreover, buffer 2.5 μl), and dNTPs, primers, and double distilled H_2_O about 0.5, 1, and 15 μl used, respectively. For the PCR, the following protocols were set up. An initial denaturation step lasted for 5 min at 95°C, 30 cycles of denaturation at 95°C for 30 s, primer annealing for 15 s at 50°C, extension for 37 s at 72°C, and the final extension for 5 min at 72°C and for *ompB* gene (Segura et al., 2023). The reaction consisted of the same protocol as had been used for the citrate synthase gene (*gltA*). After making volume, further PCR was performed with the following conditions: initial denaturation step lasted for 5 min at 95°C, followed by 30 cycles of denaturation that persisted for 30 s at 95°C; primer annealing was performed for 30 s at 53°C, and finally, extension lasted for 37 s at 72°C.

#### 2.6.4 PCR amplification of enzootic nasal tumor virus

To pinpoint an 832-bp fragment of ENTV from tick DNA, primers with the subsequent sequences were designed [(forward) 5′-CCTTGGTTCCCCAGAGAAGG-3′ and (reverse) 5′-TGGGTATTATARRCACGAGGA-3′]. The amplifications were conducted in 25 μl with 15 μl of double distilled H_2_O, 1 μl of primers, 0.5 μl of dNTPs, 2.5 μl of PCR buffer, 1 μl of MgCl_2_ 0.5 μl of enzyme, and 3 μl DNA. After making volume, the next step for PCR cycling was set up with the following protocol: initial denaturation lasted about 5 min at 95°C, followed by 30 cycles with the succeeding denaturation step for 15 s at 95°C, primer annealing for 15 s at 52°C, and the final step extension for 60 s at 72°C.

All the PCR products are kept at 4°C. Then, the amplified product was added to a 1.5% agarose gel from Biowest in Shanghai, China, using 5 μl per sample. The bands were then observed using a gel documentation system from JUNYI in Beijing, China. Following purification with the QIAquick^®^ PCR Purification Kit (Qiagen, Hilden, Germany) according to the manufacturer's recommendations, the PCR-amplified products were then transferred to Sangon Company (Guangzhou, China) for Sanger sequencing.

### 2.7 Sequence and phylogenetic analyses

The generated sequences were edited in Geneious Prime Software v.2022.01. BLAST was performed on isolated sequences, and comparisons were done on various sequences that were previously deposited in the GenBank. High similar sequences were downloaded for further phylogenetic analysis. After that, alignment was performed using CLUSTALX. Aligned sequences were checked on the basis of their same length; gaps and extra length were trimmed. Finally, after making manageable alignment, the MEGA v.11.0 software was used for making phylogenetic trees based on the maximum likelihood method (Swofford, 2003). The bootstrap consensus tree inferred from 1,000 replicates (Swofford, 2003) was taken to represent the evolutionary history of the taxa analyzed.

### 2.8 Data analysis

The data were subjected to analysis using the SPSS software (version 16). The variables were compared using chi-squared, Fisher's exact, and logistic regression tests. A *p* < 0.05 was deemed to be statistically significant.

### 2.9 Accession numbers

In the current research, different sequences were generated and submitted to the GenBank for accession numbers. Supplementary Table S1 illustrates pathogens with their accession numbers.

## 3 Results

### 3.1 Tick collection and species identification

**Figure 3:**
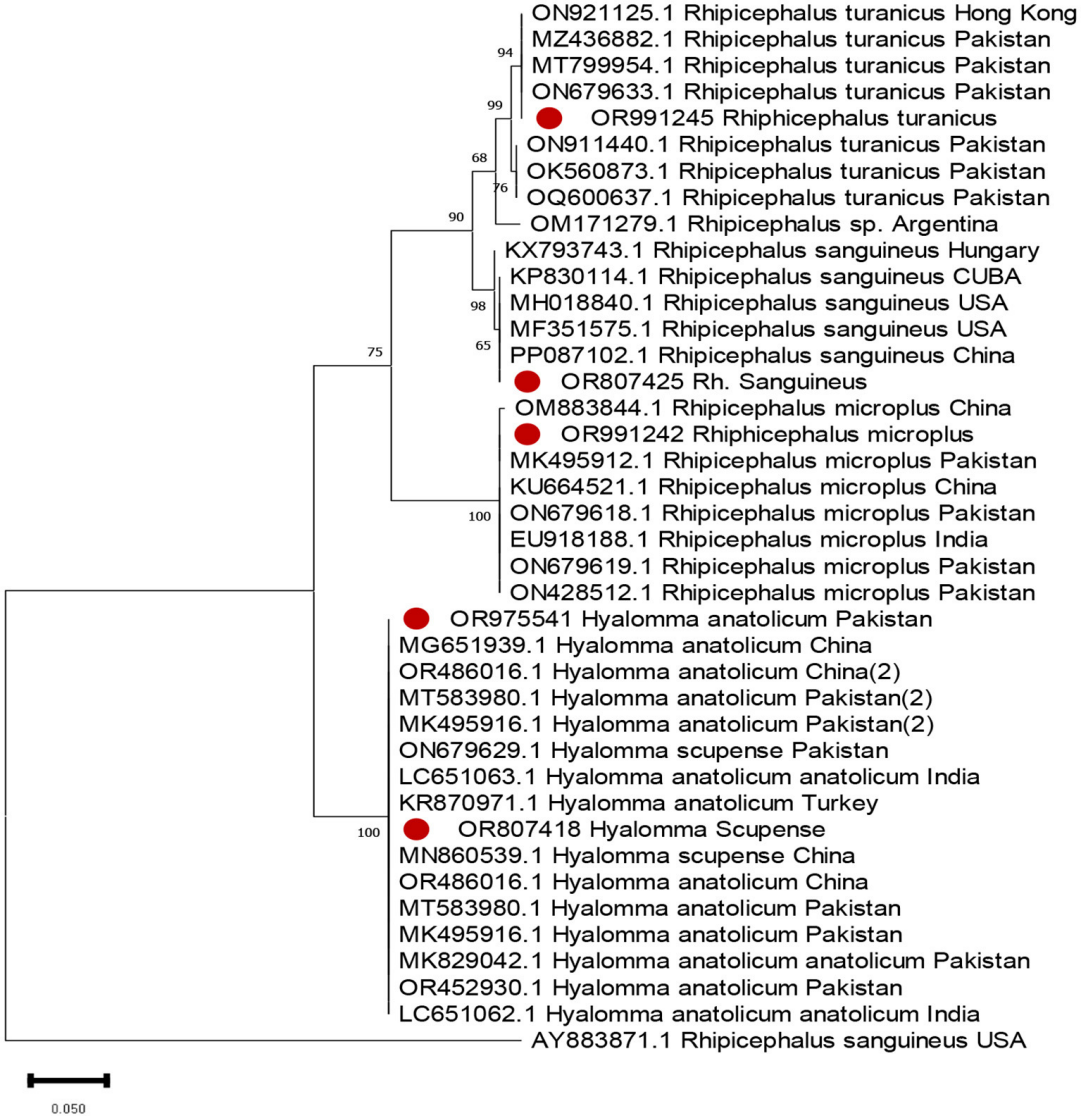
Molecular identification and phylogenetic analysis of ticks.

Both livestock and pets were examined for ticks. The animals examined included 43 cattle, 46 buffalo, 51 goats, and 44 sheep from different livestock farms. The pet animals included 21 dogs and 3 cats. Five samples were collected from poultry. In total, 213 ticks were collected from these animals. Tick samples were collected from different districts, and DNA was extracted. The ticks were identified by sequencing their rRNA sequences and aligning them with known sequences. Two other tick genera were also identified. The prevalence of these genera differed significantly. Among the collected ticks, the genus *Hyalomma* exhibited the highest prevalence, followed by *Rhipicephalus. Five* tick species were identified in this study, among which *H. anatolicum* was the most prevalent, followed by *Rh. turanicus*, with a prevalence rate of 16%. *Rhipicephalus microplus* was the third most common species, accounting for only 2% of the samples. The prevalence of *Rh. sanguineus* was found to be extremely low. Figure 3 shows the evolutionary tree based on the 16s rRNA gene of the obtained sequences. The created phylogenetic tree is divided into four different clades, and the expressed sequences are designated with red circles.

Tick species identification is based on PCR amplification, including phylogenetic analysis targeting the 16s ribosomal gene. The study sequence was analyzed for tick identification, designated with red dots.

### 3.2 Detection of tick-borne protozoa, virus, and bacterial pathogens

A comprehensive PCR analysis was conducted on 213 ticks, encompassing ticks from five distinct species, to detect the presence of prevalent tick-borne protozoan, bacterial, and viral pathogens. Of the 49 tick pools examined, 28.99% tested positive for tick-borne infections. The infection rate was determined using a comprehensive statistical analysis, yielding a calculated value of 17.45, 0.002 (Table 1). Of the 213 tick samples that were combined, 3 (1.41%) tested positive for ENTV, 11 (5.16%) tested positive for *Anaplasma*, 1 (0.47%) tested positive for *Rickettsia*, and 9 (4.23%) tested positive for *Theileria*. The prevalence of ENTV in the tick genus *Rhipicephalus* was significantly higher (1.82%) than that in other tick genera. Similarly, the bacterial pathogen *Rickettsia* had a significantly higher prevalence rate of 1 (0.97%) in the tick genus *H. anatolicum* than in the other tick genera. Additionally, the bacterial pathogen *Anaplasma* was found to have a higher prevalence of 8 (7.77%) in *Hyalomma* ticks than in other tick genera. Furthermore, *Theileria* was more prevalent in the tick genus *Rhipicephalus*. No tick-borne pathogenic DNA was detected in *Hy. scupense*.

**Table 1:** Positive rate of TBPs among different species of ticks.

**Genus**	**Species**	**Total samples**	**Positive ENTV in species**	**Positive ENTV in genus**	**Positive *Anaplasma* in species**	**Positive *Anaplasma* in genus**	**Positive *Rickettsia* in species**	**Positive *Rickettsia* in genus**	**Positive *Theileria* in genus**	**Positive *Theileria* in species**	**Total positive**
*Hyalomma*	*Hy. anatolicum*	80	1 (1.25%)	1 (1.25%)	8 (10.00%)	8 (10.00%)	1 (1.25%)	1 (1.25%)	3 (3.75%)	3 (3.75%)	26 (32.50%)
	*Hy. scupense*	23	0 (0.00%)	0 (0.00%)	0 (0.00%)	0 (0.00%)	0 (0.00%)	0 (0.00%)	0 (0.00%)	0 (0.00%)	0 (0.00%)
*Rhipicephalus*	*Rh. turanicus*	70	2 (2.86%)	2 (2.86%)	2 (2.86%)	3 (4.29%)	0 (0.00%)	0 (0.00%)	4 (5.71%)	6 (8.57%)	19 (27.14%)
	*Rh. sanguineus*	5	0 (0.00%)	0 (0.00%)	0 (0.00%)	0 (0.00%)	0 (0.00%)	0 (0.00%)	0 (0.00%)	0 (0.00%)	0 (0.00%)
	*Rh. microplus*	35	0 (0.00%)	0 (0.00%)	1 (2.86%)	0 (0.00%)	0 (0.00%)	0 (0.00%)	2 (5.71%)	0 (0.00%)	3 (8.57%)
Total		213	3 (1.41%)	3 (1.41%)	11 (5.16%)	11 (5.16%)	1 (0.47%)	1 (0.47%)	9 (4.23%)	9 (4.23%)	48 (22.54%)
Chi-square			1.97^NS^	1.97^NS^	6.49^NS^	7.36^NS^	1.67^NS^	1.67^NS^	1.86^NS^	6.09^NS^	17.45^**^
*P*-value			0.741	0.741	0.166	0.118	0.796	0.796	0.762	0.192	0.002

NS, non-significant (P < 0.05); ^*^significant (P > 0.05); ^**^highly significant (P < 0.01).

### 3.3 Distribution of tick-borne pathogens among districts and provinces

After surveying the positivity rate of TBPs among different ticks, we evaluated the distribution of positive ticks among different districts, which is important for evaluating risk in terms of spatial distribution. First, we compared the distribution of TBP-positive rates among different provinces. As shown in Table 2, the positive rates between the provinces differed significantly (or non-significantly). Samples were collected from several districts in the provinces of Sindh and Punjab. Although different rates of TBPs were identified, no significant differences were observed between the districts of the two provinces. The higher the positivity rate of TBPs, the greater the risk. Therefore, provinces with higher positivity rates face a higher risk of developing TBPs.

**Table 2:** Positive rate of TBPs among districts and provinces.

**Provinces**	**Districts**	**Samples to be tasted**	**ENTV Positive among districts**	***Anaplasma* Positive among districts**	***Theileria* positive among districts**	***Rickettsia* positive among districts**	**Total positive**
Sindh	Nawab shah	19	3 (15.79%)	6 (31.58%)	3 (15.79%)	0 (0.00%)	12 (63.16%)
	Karachi	33	0 (0.00%)	0 (0.00%)	2 (6.06%)	0 (0.00%)	2 (6.06%)
	Hyderabad	32	0 (0.00%)	0 (0.00%)	1 (3.13%)	0 (0.00%)	1 (3.13%)
Punjab	Lodhran	25	0 (0.00%)	7 (28.00%)	8 (32.00%)	0 (0.00%)	15 (60.00%)
	Sahiwal	12	0 (0.00%)	1 (8.33%)	0 (0.00%)	0 (0.00%)	1 (8.33%)
	Okara	12	0 (0.00%)	6 (50.00%)	2 (16.67%)	0 (0.00%)	8 (66.67%)
	Mianwali	20	0 (0.00%)	9 (45.00%)	7 (35.00%)	0 (0.00%)	16 (80.00%)
	Kabola	8	0 (0.00%)	1 (12.50%)	1 (12.50%)	0 (0.00%)	2 (25.00%)
	Pakpattan	8	0 (0.00%)	0 (0.00%)	0 (0.00%)	0 (0.00%)	0 (0.00%)
	Faisalabad	10	1 (10.00%)	0 (0.00%)	0 (0.00%)	0 (0.00%)	1 (10.00%)
Kashmir	Bagh	10	0 (0.00%)	0 (0.00%)	3 (30.00%)	4 (40.00%)	7 (70.00%)
	Mirpur	14	0 (0.00%)	0 (0.00%)	0 (0.00%)	0 (0.00%)	0 (0.00%)
Baluchistan	Quetta	10	0 (0.00%)	0 (0.00%)	0 (0.00%)	0 (0.00%)	0 (0.00%)
Total		213	4 (1.88%)	30 (14.08%)	27 (12.68%)	4 (1.88%)	65 (30.52%)
Chi-square			27.06^**^	56.92^**^	32.27^**^	82.75^**^	97.21^**^
*P*-value			0.008	0.000	0.001	0.000	0.000

NS, non-significant (P < 0.05); ^*^significant (P > 0.05); ^**^highly significant.

### 3.4 Distribution of tick-borne pathogens among animals

After analyzing the positivity rate among different districts and ticks, we analyzed the TBP positivity rates among different species of livestock. As shown in Table 3, the positivity rate of TBPs was significantly different among the tested animals (χ^2^ = 58.66, *P* = 0.000). Among the animal species, goats had the highest positive rate, and all four types of bacteria, virus, and protozoan pathogens were found in the ticks from goats, followed by sheep (21, 47.73%), cattle (4, 9.30%), and buffalo (3, 6.52%). The prevalence of *Theileria* and *Anaplasma* was high. In the case of cats, one (33.33%) *Rickettsia* species was identified, and in poultry, one (20.00%) *Anaplasma* species was detected. Combined with the distribution of ticks from different animals, these results indicate that TBPs are differentially distributed between different species of ticks and animals.

**Table 3:** Positive rate of TBPs among animals.

**Animals**	**Samples to be tasted**	**Positive for ENTV**	**Positive for *Anaplasma***	**Positive for *Theileria***	**Positive for *Rickettsia***	**Total positive**
Goat	51	3 (5.88%)	12 (23.53%)	12 (23.53%)	3 (5.88%)	30 (58.82%)
Sheep	44	4 (9.09%)	11 (25.00%)	6 (13.64%)	0 (0.00%)	21 (47.73%)
Cattle	43	0 (0.00%)	2 (4.65%)	2 (4.65%)	0 (0.00%)	4 (9.30%)
Buffalo	46	0 (0.00%)	1 (2.17%)	2 (4.35%)	0 (0.00%)	3 (6.52%)
Poultry	5	0 (0.00%)	1 (20.00%)	0 (0.00%)	0 (0.00%)	1 (20.00%)
Dog	21	0 (0.00%)	0 (0.00%)	0 (0.00%)	0 (0.00%)	0 (0.00%)
Cat	3	0 (0.00%)	0 (0.00%)	0 (0.00%)	1 (33.33%)	1 (33.33%)
Total	213	7 (3.29%)	27 (12.68%)	22 (10.33%)	4 (1.88%)	60 (28.17%)
Chi-square		9.76^NS^	22.27^**^	16.73^*^	23.59^**^	58.66^**^
*P*-value		0.135	0.001	0.010	0.001	0.000

NS, non-significant (P < 0.05); ^*^significant (P > 0.05); ^**^highly significant (P < 0.01).

### 3.5 Phylogenetic analysis of TBPs

**Figure 4:**
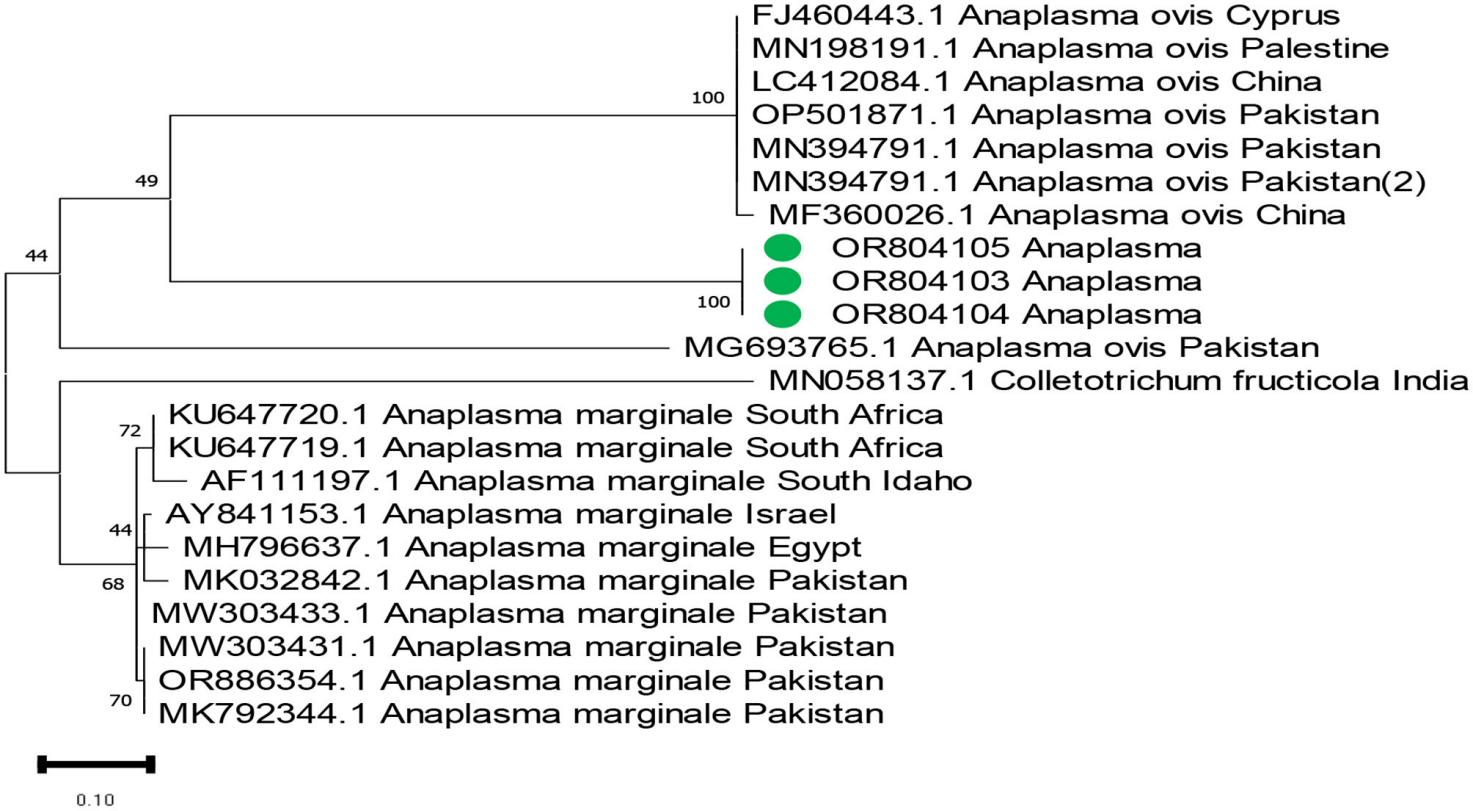
Phylogenetic tree based on the 16s rRNA of *Anaplasma marginale* partial sequence. Targeted sequences are expressed in green circles. Supporting bootstrap values were used, and *Anaplasma marginale* from Pakistan was set up as an outgroup.

**Figure 5:**
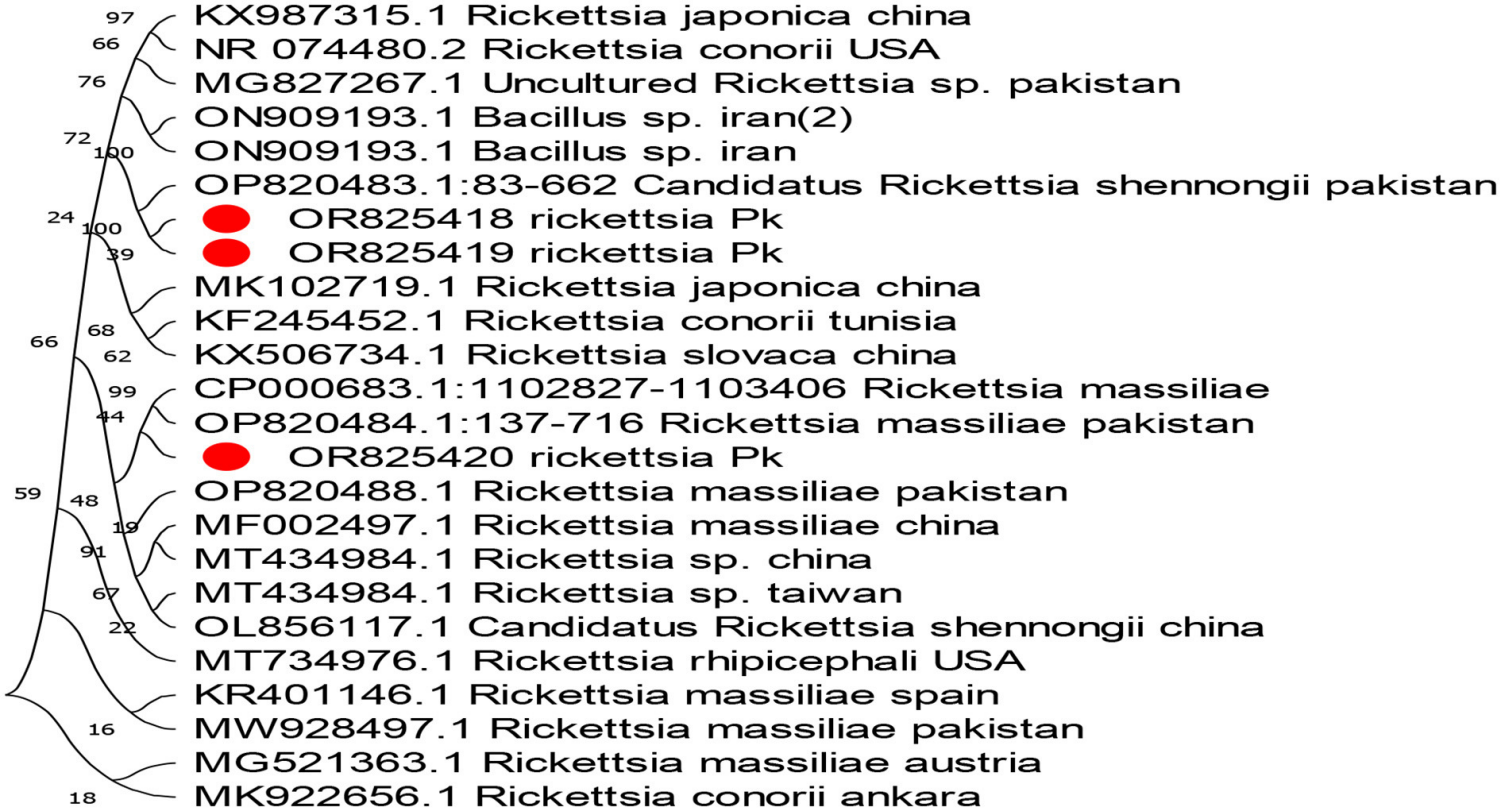
Evolutionary tree based on the outer membrane surface protein sequence of *Rickettsia*. The bootstrap values were kept standard, and substitutions were based on scale bar values. As an outgroup, *Rickettsia conorii* from Ankara and *Rickettsia massiliae* were used. The obtained sequences are expressed in red circles.

**Figure 6:**
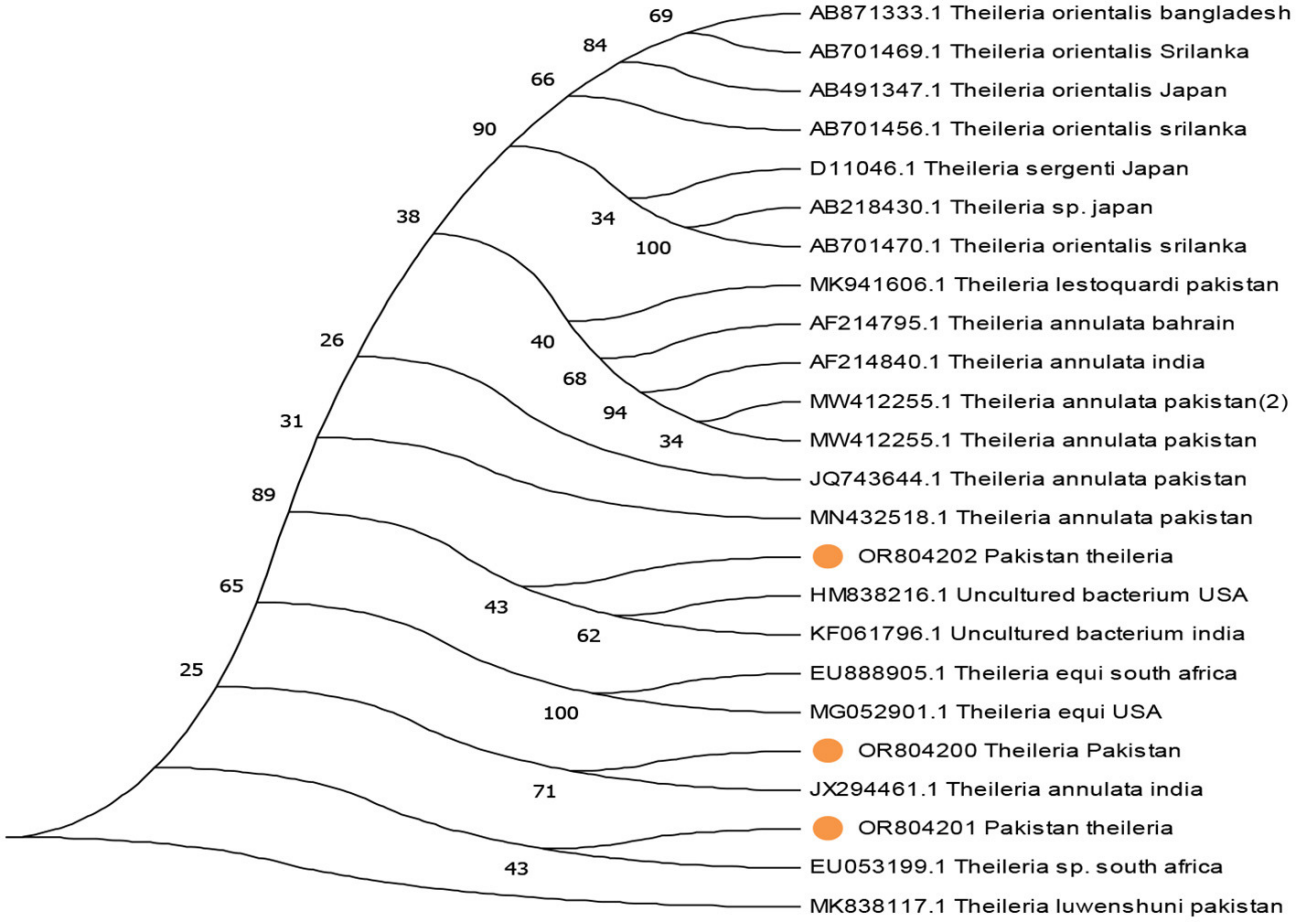
Evolutionary tree based on the 18s rRNA of *Theileria*, with the *Theileria luwenshuni* used as an outgroup. Standard values of bootstrap considered along with the scale bar stipulate substitution at each branch site. The extracted sequences are expressed in yellow circles.

**Figure 7:**
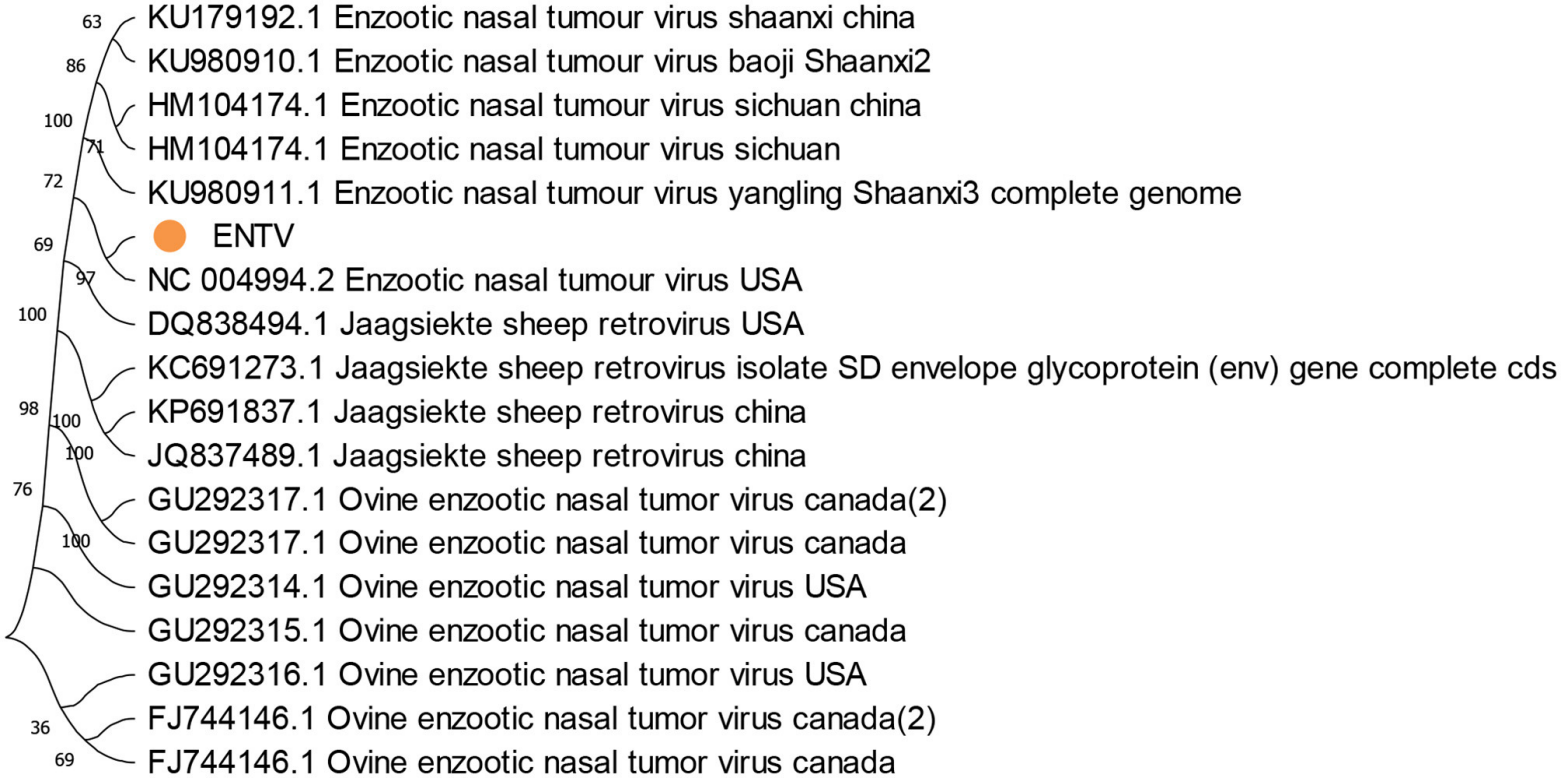
Evolutionary tree of the whole-genome sequence analysis of ENTV obtained from goats in Pakistan. The obtained genomes are expressed in yellow circles and shown in the middle of the figure. The ovine enzootic nasal tumor virus in Canada was set as an outgroup. The standard bootstrap value was used, and the maximum likelihood method was applied for evolutionary tree construction.

The phylogenetic analysis of *Anaplasma marginale, Rickettsia canadensis, Theileria*, and ENTV delineates our findings in Figures 4–7. After completing all three *Anaplasma marginale* sequences, it has been documented that all three partial sequences formed a different clade and were partially related to other sequences from China and Pakistan (Figure 4). The results of the phylogenetic analysis based on the outer surface protein of *Rickettsia canadensis* showed a close clustering pattern close to two sequences, specifically OR825418 and OR825419, which were obtained from different ticks. These sequences exhibited a close clustering pattern with sequences from Pakistan, China, and Iran. The sequences acquired from GenBank were derived from various tick species. The sequence with the identifier OR8254820 was identified to form a separate clade, as shown in Figure 5. The partial sequences of *Theileria* of different ticks, including *Hyalomma* and *Rhipicephalus*, were collected from various locations in Pakistan, such as Punjab, Sindh, Balochistan, and AJK. Phylogenetic analysis of three partial *Theileria* sequences revealed distinct clades from other sequences in GenBank. *Theileria* (OR804202) is similar to sequences from the United States and India. *Theileria* (OR804200) showed a close relationship to sequences from India. Furthermore, *Theileria* (OR804201) showed 100% similarity with a South African isolate (Figure 6).

The phylogenetic analysis of the current whole-genome sequence of ENTV isolated from *Rh. Turanicus* and *Hy. anatolicum* (OR991120) in sheep and goats from various districts of Pakistan revealed a relatively high homology (100%) with ENTV from the United States (NC004994). The lowest divergence ratios (30%) were detected with ENTV isolated from goats in Canada (FJ744146) (Figure 7).

## 4 Discussion

In this study, 213 tick species from two genera, namely, *Rhipicephalus* and *Hyalomma*, with five species ascertained, namely, *R. turanicus, R. sanguineus, R. microplus, Hy. anatolicum*, and *Hy. scupense*, were assembled from the Punjab, Balochistan, Kashmir, and Sindh provinces in Pakistan. According to these studies, the provinces that share common borders with Afghanistan are inaugurating delving ticks in Pakistan. Our finding proclaimed that nine (4.23%) clarify that *Theileria* were detected in all the surveyed tick species, except *Hy. scupense* and *R. sanguineus*. *Theileria* infections have been investigated using various techniques in different regions worldwide, including Pakistan. For example, in Egypt, the prevalence of *T. annulata* in buffaloes is 15.49% (El-Ashker et al., 2015). Few reports concluded that in Pakistan, the prevalence of *T. annulata* was reported to be 16.3% and 29.9% in ruminants (Zeb et al., 2020). Our findings align with worldwide research, including those in Pakistan, with a slight difference in the prevalence rate. Differences in prevalence could be due to changes in temperature, humidity, agriculture methods, and vector populations across different locations globally (Zafar et al., 2022) with a high prevalence in the southern part of Lahore district of Punjab Province, where *Theileria* was detected to be 65% prevalent in both *Rhipicephalus* and *Hyalomma* in sheep (Durrani et al., 2011). Research conducted in the following countries, Turkey, Pakistan, and China, indicated that *Theileria* has been detected to be the most abundant species, with a prevalence rate of 34.56%, 79%, and 78%, respectively (Durrani et al., 2011). Besides, the *Theileria* prevalence rate in the present study is lower than the previous studies, which detected *Theileria* from various mammalian hosts in preference to ticks; hence, the results were found to be inconsistent with our findings. In addition, the ascertained three partial 18s rRNA sequences of *Theileria* are highly pathogenic in nature due to their correspondence with other *Theileria* spp., which were determined from different regions in Pakistan.

In this study, *A. marginale* was detected in 8 (10.00%) *Hy. anatolicum* species and 3 (4.29%) *R. turanicus* species using molecular methods. Few reports suggest that 20 different kinds of ticks are the reservoir host of *A. marginale*, which leads to oppressive complications, for instance, bovine anaplasmosis (Pothmann et al., 2016). In Pakistan, conventional methods such as microscopy analysis are used for detecting *A. marginale*; molecular approaches for detecting such a pathogen in ticks are meager (Khan et al., 2017). The results revealed that *A. marginale* is dominant in *Hy. anatolicum*. Our finding is contrary to the study that identified *R. microplus* as a consequential host for spreading *A. marginale* in various developing countries (Kocan et al., 2010). Our finding contradict the report of Ramzan et al., whose verified research in Multan districts proclaimed the prevalence of *A. marginale* in cattle and buffalo (Ramzan et al., 2020). Our results suggested that small ruminants are utterly more infected with *A. marginale* than large ruminants, which is in accordance with the discrepancy in the susceptibility of cattle and buffalo breeds to anaplasmosis (Sajid et al., 2014).

Our findings concluded that *R. canadensis* was detected in 1 (1.25%) *Hy. anatolicum* tick collected from cats and domestic goats. Candidatus *Rickettsia*, an agent of unknown pathogenicity, is a common vector in cat fleas (Mediannikov et al., 2015; Legendre and Macaluso, 2017). SFGR infections in cats could also be caused by tick-borne SFGR (Jabbar et al., 2015). These findings are contrary to other research conducted in the tribal areas of Rawalpindi, Pakistan, showing that *Rickettsia* is predominant in different tick species such as *Rh. turanicus, Rh. microplus, Rh. haemaphysaloides, and Rhipicephalus* species (Ali et al., 2021). Few investigations have reported that *Rh. sanguineus, Rh. sulcatus, Rh. lunulatus, Rh. muhsamae*, and *Rh. senegalensis* are the reservoir hosts of this pathogen (Eremeeva et al., 2006).

Our findings suggested that ENTV was detected in 2 (2.86%) *Rh. turanicus* and 1 (1.25%) *Hy. anatolicum* ticks were collected from a total of 3 (5.88%) small ruminant goats and 4 (9.09%) sheep in the Nawabshah and Faisalabad districts of Pakistan. In Pakistan, there are no reports of ticks infesting ruminants equated with ENTV infection, and to our knowledge, this is the first report of ENTV associated with small ruminant ticks. Research has concluded that ENTV isolated from goats and sheep belongs to ENTV-1 and ENTV-2, respectively (Ortín et al., 2003). Oncogenic retroviruses transform epithelial cells through their envelope (Monot et al., 2015). ENTV may cause severe complications in animals in terms of clinical manifestations such as dry and productive cough, exudative nasal discharge, difficulty breathing, stretching neck breathing, and ultimately facial deformities (De las Heras et al., 1995). Gradually, the animals lose weight and die. A small ruminant population is highly affected by this tumor worldwide; most recent cases have been reported in Turkey (Ajayi et al., 2013; Özmen and Serpin, 2016).

There is a varying route of ENTV transmission among animals, but the most common exploration route is the respiratory route via nasal and cough exudate. It has been reported that ENTV is reproduced experimentally by nebulization (Walsh et al., 2013). Other routes of transmission, such as milk, colostrum, or intrauterine or perinatal infections, as have been reported for JSRV and ENTV, should also be considered (Caporale et al., 2005; Grego et al., 2008). Infected animals are the more common cause of ENTV transmission among disease-free flocks.

Our findings showed that 3 (6.52%) buffaloes, 4 (9.30%) cattle, 30 (58.82%) goats, 21 (47.73%) sheep, 1 (20.00%) poultry, 1 (33.33%) cat, and 21 (0%) dogs were infested with TBPs. Cattle comprised the highest proportion of livestock infested with TBPs. This may be due to the thin skin and other supporting conditions for the growth of ticks in Pakistan, like temperature, humidity, and habitat (Ghafar et al., 2020). Our findings indicate lower rates of tick infestation in buffaloes than in cattle, which aligns with previous research that also found comparable findings (Ghafar et al., 2020; Ramzan et al., 2020; Hussain S. et al., 2023).

Sheep have a lower TBPs infestation rate than goats. Several studies conducted in Pakistan have consistently demonstrated a prevalent trend of tick infestation in livestock, revealing that sheep manifest a lower infection rate (11.1%) than goats (60.0%) (Alessandra and Santo, 2012). In contrast, another study reported that infestation was higher in sheep than in goats (Rashid et al., 2018; Ramzan et al., 2019). The main reason for low infestation may be the presence of wool on the sheep's body. Wool may be a better protective factor against tick attachment in sheep. Our results agree with those of previous findings on tick infestation (Atif, 2012; Monfared et al., 2015).

Our findings among various districts concluded that Mianwali, Lodhran, and Okara in Punjab Province are more highly affected by TBPs than other districts due to the humid environmental conditions, as most of the rice crop is reared in that area (Ashraf et al., 2021). The maximum levels of egg-laying and hatching of Hyalomma ticks are observed at 32–34°C with 85% humidity (Durrani and Shakoori, 2009).

## 5 Conclusion

In the present study, we performed an etiological survey of tick-borne pathogens among livestock and pet animals in Pakistan. The results showed that various tick-borne pathogens were identified in both livestock and pet animals, and these pathogens are greatly differentially distributed among ticks and animals, which implied a risk for both animals and humans. Furthermore, a new virus, ENTV, was identified in animals in Pakistan. Further studies involving a larger number of ticks sampled across the country and detailed transmission studies should be conducted to further evaluate the prevalence of tick-borne pathogens of veterinary and medical importance.

## Data availability statement

The datasets presented in this study have been deposited to the NCBI Genebank Repository. The accession numbers can be found in the Supplementary material.

## Ethics statement

The animal studies were approved by the Research Institute of Veterinary Medicine in Shenyang Agriculture University. The studies were conducted in accordance with the local legislation and institutional requirements. Written informed consent was obtained from the owners for the participation of their animals in this study.

## Author contributions

AJ: Conceptualization, Writing—original draft, Writing—review & editing, Data curation, Formal analysis, Funding acquisition, Investigation, Methodology, Project administration, Resources, Software, Supervision, Validation, Visualization. ZY: Software, Writing—original draft, Writing—review & editing. YW: Data curation, Formal analysis, Writing—original draft, Methodology. QX: Methodology, Writing—review & editing. MA: Methodology, Writing—review & editing. SG: Data curation, Formal analysis, Methodology, Writing—review & editing. XH: Conceptualization, Data curation, Formal analysis, Funding acquisition, Investigation, Methodology, Project administration, Resources, Software, Supervision, Validation, Visualization, Writing—review & editing, Writing—original draft. ZC: Conceptualization, Data curation, Formal analysis, Funding acquisition, Investigation, Methodology, Project administration, Resources, Software, Supervision, Validation, Visualization, Writing—original draft, Writing—review & editing.
