# Supplementary material for: Tick-borne Rickettsia, Anaplasma, Theileria, and enzootic nasal tumor virus in ruminant, PET, and poultry animals in Pakistan
Source: Front Microbiol. 2024 Mar 26;15:1359492. doi: 10.3389/fmicb.2024.1359492 (PMC11002113; doi:10.3389/fmicb.2024.1359492)
Supplement: Supplementary file 1 [file Table_1.docx]

**Table S1 Genebank accession Numbers**

| **Pathogen** | **Accession Number** |
| --- | --- |
| *Hyalomma anatolicum* | OR975541 |
| *Hyalomma scupense* | OR807418 |
| *Rhipicephalus turanicus* | OR991245 |
| *Rhipicephalus microplus* | OR991242 |
| *Rhipicephalus sanguineus* | OR807425 |
| *Theileria Seq 1* | OR804200 |
| *Theileria Seq 2* | OR804201 |
| *Theileria Seq 3* | OR804202 |
| *Anaplasma marginalee seq 1* | 0R804103 |
| *Anaplasma marginale seq 2* | OR804104 |
| *Anaplasma marginale seq 3* | OR804105 |
| *Rickettsia seq 1* | OR825418 |
| *Rickettsia seq 2* | OR825419 |
| *Rickettsia* seq 3 | OR825420 |
| Enzooric Nasal tumor Virus | OR991120 |
